# Acceptance, Drivers, and Barriers to Use of mHealth Apps to Improve Quality of Life in Female Patients Affected by Hypothyroidism: Cross-Sectional Study

**DOI:** 10.2196/67317

**Published:** 2025-08-07

**Authors:** Moritz Doll, Ranujan Chandrakumar, Lisa Maria Jahre, Eva-Maria Skoda, Hannah Dinse, Dagmar Führer, Eleni Lampropoulou, Martin Teufel, Alexander Bäuerle

**Affiliations:** 1 Clinic for Psychosomatic Medicine and Psychotherapy, LVR-University Hospital Essen, University of Duisburg-Essen, Virchowstr. 174, Essen, 45147, Germany, 0049 201438755203; 2 Center for Translational Neuro- and Behavioral Sciences (C-TNBS), University of Duisburg-Essen, Essen, Germany; 3 Department of Endocrinology, Diabetes and Metabolism, University of Duisburg-Essen, University Hospital Essen, Essen, Germany

**Keywords:** hypothyroidism, mHealth, acceptance, unified theory of acceptance and use of technology, quality of life, digital health, chronic disease, female patients, cross-sectional study, mobile health, mobile phone

## Abstract

**Background:**

Hypothyroidism is a common chronic disease that can substantially impair physical and mental well-being and is associated with lower quality of life, a trend that interventions delivered by mobile health (mHealth) apps could ameliorate.

**Objective:**

The objective of this study was to evaluate the acceptance and its influencing predictors of mHealth interventions in female patients affected by hypothyroidism to improve their quality of life. The focus on female patients reflects the significantly higher prevalence of hypothyroidism in women and their underrepresentation in many prior studies on technology acceptance and mHealth use.

**Methods:**

A survey-based, cross-sectional study, which included 318 female patients affected by hypothyroidism (assessed via self-reported diagnosis according to International Classification of Diseases-10 criteria, aged 18 y or older), was conducted online between April 2023 and April 2024 in Germany. Participants were recruited via local and online self-help groups, social media platforms, and medical practices using flyers. Sociodemographic, health, and eHealth–related data were assessed. To determine acceptance and its drivers and barriers, an extended version of the unified theory of acceptance and use of technology (UTAUT) model was applied. Group comparisons (*t* tests, ANOVAs) and multiple hierarchical regression analyses were conducted. Only complete datasets were included in the analysis.

**Results:**

Acceptance of mHealth apps was high (mean 4.10, SD 0.91), with 76.1% (n=242) of the participants reporting high acceptance, 18.6% (n=59) reporting moderate acceptance, and only 5.3% (n=17) reporting low acceptance. Significant predictors of acceptance were place of residence: medium-sized city (β=0.34; *P*=.02) and small town or rural area (β=0.28; *P*=.003), fatigue (β=0.54; *P*<.001), internet anxiety (β=−0.20; *P*=.002), and the UTAUT predictors effort expectancy (β=0.37; *P*<.001), performance expectancy (β=0.32; *P*<.001), and social influence (β=0.20; *P*<.001). The extended model explained 56.1% of the variance in acceptance.

**Conclusions:**

The high level of acceptance of mHealth apps observed among female patients affected by hypothyroidism indicates that mHealth interventions can provide such patients with valuable support to manage the disease and improve their quality of life. Addressing drivers and barriers of acceptance will be crucial for the successful implementation of mHealth interventions in hypothyroidism management, for example, by mHealth developers, clinicians, or policy makers. These include intuitive and accessible design (effort expectancy), clear communication of app benefits (performance expectancy), and fostering health care professional support (social influence), while addressing barriers such as internet anxiety. The study also contributes to advancing gender-sensitive mHealth research by applying the UTAUT model to this patient group.

## Introduction

### Background

Due to their high prevalence, large parts of the health care system are involved in treating and managing thyroid diseases, as about 0.2% to 5.3% in Europe, 0.3% and 3.7% in the United States, and approximately 3% worldwide are affected by hypothyroidism [1,2]. In Germany, 8.9% of female and 1.8% of male patients are diagnosed with a thyroid disorder [3]. Among all population groups, female and older adults have an especially elevated risk of thyroid disease [4-7].

For patients, thyroid disease can have a wide range of consequences, especially for physical and mental health. Frequent symptoms of overt hypothyroidism include obesity, fatigue, infertility or subfertility, and various cardiovascular symptoms such as bradycardia [8,9]. Thyroid hormone imbalance is further associated with mental health comorbidities, including depression and anxiety, particularly when untreated [10-13], and is overall linked to a decreased quality of life [14].

Treating hypothyroidism typically involves thyroid hormone replacement therapy with levothyroxine [15]. Such therapy requires administering the correct dose of levothyroxine, which can be determined and aggravated by various factors, including body weight, etiology of hypothyroidism, age, and clinical context [15]. Underdosing leaves patients hypothyroid, while overdosing may induce iatrogenic hyperthyroidism [16].

However, even if the correct dose of levothyroxine is administered, as evidenced by normalized serum thyroid stimulating hormone levels, about 10%‐15% of the patients remain dissatisfied due to persistent symptoms of hypothyroidism [15,17-19]. Moreover, studies report that only 59% of patients prescribed levothyroxine were fully adherent [20], and 22% (72/327) of participants in another study reported not adhering to thyroxine replacement therapy [21]. Underlying reasons for nonadherence have included forgetfulness and carelessness, and non- or poor adherence has also been associated with a lower level of education, long-term therapy (ie, >12 months) with levothyroxine, and unattended medical appointments, as investigated in a study [22]. Concerns about medication, as well as affordability and accessibility, are additional factors that influence adherence [23]. The wide range of symptoms and comorbidities, along with the challenges in adjusting therapy associated with poor compliance, underscore the need to support patients with thyroid disease at higher levels.

In recent years, mobile health (mHealth) apps have emerged within the health care systems and have been the subject of diverse studies. Since more than 4.7 billion people worldwide use smartphones, mHealth offers a widely accessible, cost-effective, and sustainable way of managing chronic diseases [24-26]. Improving diagnostics by using symptom checkers, implementing interventions for behavioral changes, improving medication adherence, and providing “disease-related education,” as well as facilitating digital exchanges with health care providers, showcases the value of mHealth apps in managing chronic diseases [24,27]. For patients with thyroid disorders, the benefits are numerous. One study evaluating the mHealth app “BOOST Thyroid App” revealed an increase in health literacy, improved patient-doctor interaction (including fewer or shorter appointments), and enhanced overall quality of life after using the mHealth app [28]. In the study, 95.8% of patients reported that the mHealth solution generally supported them in managing their disease [28]. Overall, these study results clarify the usefulness of mHealth interventions in supporting patients with thyroid disease.

However, implementing mHealth interventions in routine care presents several obstacles, including concerns about data privacy, trust in online information, and smartphone access [29,30]. Older adults, in particular, tend to be insufficiently experienced with mHealth apps or may not be aware of their existence, and the lack of face-to-face communication presents a barrier for older people [31].

To sustainably implement mHealth apps for patients affected by hypothyroidism despite these challenges, the acceptance of mHealth apps and influencing factors need to be investigated. However, at present, no study has evaluated the acceptance of mHealth interventions to improve the quality of life of female patients affected by hypothyroidism by using acknowledged measurement methods. This study focuses on female patients with hypothyroidism for various reasons: first, since hypothyroidism is significantly more prevalent among women than men, the acceptance of mHealth interventions by female patients is of major clinical importance [5,6]. Second, female patients have historically been underrepresented in clinical and digital health research, which can lead to gender bias in the development and implementation of health technologies [32,33]. Addressing this gap aligns with the principles of gender medicine and ensures that mHealth solutions are developed based on evidence that adequately reflects the experiences and needs of female patients with hypothyroidism [34]. Even so, several variables to determine the acceptance of technology can be approached by the unified theory of acceptance and use of technology (UTAUT) [35], including the following variables: performance expectancy (PE), effort expectancy (EE), social influence (SI), facilitating conditions, and behavioral intention (BI) to use (ie, operationalization of acceptance) new technology [36-38]. Regarding mHealth (eg, mHealth interventions) apps in particular, various studies have also shown the usefulness of the UTAUT in investigating those variables [38-41].

### Objectives

This study aimed to investigate the acceptance of mHealth interventions among female patients affected by hypothyroidism and factors contributing to such acceptance as a means of improving their quality of life. In addition, questions regarding the lack of mHealth interventions implemented in consideration of their acceptance and contributing factors of such acceptance have thus far remained unanswered. In doing so, we used an extended UTAUT model. The additional variables capture sociodemographic and medical issues, as well as information and communication technology–related data, that is, the technical basis of communication via mHealth apps.

This study focuses on the following questions: (1) What is the general level of acceptance of mHealth apps among female patients affected by hypothyroidism? (2) To what extent do female patients affected by hypothyroidism differ in their acceptance regarding sociodemographic and medical factors? and (3) What are the key facilitators and obstacles affecting the acceptance of mHealth apps?

## Methods

### Study Design

A survey-based, cross-sectional study was conducted to assess the acceptance of mHealth apps to improve the quality of life of female patients affected by hypothyroidism. From April 2023 to April 2024, participants were primarily recruited via local and analog self-help groups (eg, Schilddrüsenliga e.V.) and social media platforms (eg, Facebook), supplemented by recruitment through medical practices using a flyer containing concise information regarding the study’s background and responsible institutions. The flyer included a QR code and a link directing to the web-based questionnaire. The survey was conducted on the platform Unipark (Tivian XI GmbH).

### Ethical Considerations

All participants electronically provided their informed consent prior to the start of the survey. Participation in the survey was voluntary, anonymous, and without compensation. The study was conducted in accordance with the Declaration of Helsinki and was approved by the Ethics Committee of the Medical Faculty of the University of Duisburg-Essen (19-89-47-BO).

### Procedures and Participants

On average, completing the questionnaire took approximately 17 (SD 8) minutes. Initially, 532 participants started processing the questionnaire, of which 362 participants completed the survey, representing a 68.1% completion rate. Participants who were female, consented to participate, had a self-reported diagnosis of hypothyroidism (according to World Health Organization International Classification of Diseases-10: E03 and E89.0 [42]), legal age of 18 years or older, had sufficient German-language skills, and had internet access were eligible to participate. Only participants who completed the full assessment were included. Thus, 44 (12.2%) participants were excluded due to not meeting the inclusion criteria. Therefore, 318 (59.8%) participants were included in the final data analysis. To maintain the required high methodological standards, the STROBE (Strengthening the Reporting of Observational Studies in Epidemiology) Statement checklist (Checklist 1) was applied [43].

### Assessment Instruments

#### Overview

The questionnaire sought to gather sociodemographic, medical, and eHealth-related data. The acceptance of eHealth interventions among female patients affected by hypothyroidism and its drivers and barriers were determined by using a modified version of the UTAUT model.

#### Sociodemographic and Medical Data

Sociodemographic data gathered included age, gender, marital status, level of education, occupational status, and place of residence (population size). To gauge the participants’ health status, we assessed the existence of hypothyroidism and its symptoms by self-report. In addition, participants were asked whether they had received hormone replacement therapy, for example, with levothyroxine in the past or were currently receiving it and might have been affected by therapy’s side effects. Participants were also asked whether they had previously undergone surgery as a reason for their hypothyroidism. Physical health, mental health, and quality of life were each self-rated on a numeric rating scale from 0 to 10, with lower scores indicating lower levels of the aforementioned constructs.

#### eHealth-Related Data

Next, the participant’s eHealth literacy was assessed using the revised German version of the eHealth Literacy Scale [44], with responses given on a 5-point Likert scale (1=strongly disagree to 5=strongly agree). Its internal consistency was excellent (Cronbach α=0.92). Participants also assessed their digital confidence by rating three items on a 5-point Likert scale (1=very insecure to 5=very confident) [45-47]. Its internal consistency was excellent (Cronbach α=0.91). Next, digital overload was assessed with three items (eg, “I feel burdened by the constant availability via cell phone or email”) [45,48,49], with answers given on a 5-point Likert-type scale (1=does not apply to 5=does fully apply). The internal consistency was acceptable (Cronbach α=0.77). Last, internet anxiety was assessed using three items (eg, “I am afraid I might make an irreversible mistake when using the internet”) [40,47,48,50,51]. Again, answers were given on a 5-point Likert-type scale (1=does not apply to 5=does fully apply). The internal consistency was acceptable (Cronbach α=0.79).

#### UTAUT Predictors and Acceptance

To assess the acceptance of mHealth apps and their influencing factors, we used a modified UTAUT questionnaire [36,38]. The modified UTAUT questionnaire contained 14 items—4 assessing BI, 3 assessing SI, 3 assessing PE, and 3 assessing EE—and answers were given on a 5-point Likert scale (1=strongly disagree to 5=strongly agree). Internal consistency ranged from acceptable to excellent (Cronbach α=0.9 for BI; Cronbach α=0.77 for SI; Cronbach α=0.82 for PE; and Cronbach α=0.82 for EE).

### Statistical Analyses

Statistical analyses were performed using R (version 4.3.8; R Core Team), whereas sum scores were calculated for eHealth literacy using the revised German version of the eHealth Literacy Scale, and mean scores were calculated for digital confidence, internet anxiety, digital overload, and UTAUT scales (BI, EE, PE, and SI). Acceptance was operationalized as BI and was further categorized in accordance with prior research [45,47,52], such that scores from 1 to 2.34 indicated low acceptance, scores from 2.35 to 3.67 indicated moderate acceptance, and scores from 3.68 to 5 indicated high acceptance. Descriptive statistics were applied for sociodemographic, medical, and eHealth-related data.

Differences in acceptance based on sociodemographic and medical variables were examined with independent 2-tailed *t* tests and ANOVAs. Bonferroni correction was applied to adjust *P* values for multiple comparisons, and Levene’s test indicated homoscedasticity. Due to the given sample size, normal distribution of residuals was assumed.

Multiple hierarchical regression analysis was conducted to examine drivers of and barriers to the acceptance of mHealth apps among female patients affected by hypothyroidism. Predictors were included block-wise: (1) sociodemographic data, (2) medical data, (3) eHealth-related data, and (4) UTAUT predictors. The variance inflation factor was used to verify the absence of multicollinearity (all variance inflation factor values <2.0). A visual inspection of QQ-plots of the residuals showed no signs of violations against normality; therefore, the normal distribution of the residuals was assumed. Scatter plots of the standardized residuals and the adjusted predicted values verified homoscedasticity. Only complete datasets were included in the analysis, so there was no missing data. The level of significance was set to α<.05 for all tests. Effect sizes were reported according to Cohen, with values around 0.2, 0.5, and 0.8 indicating small, medium, and large effects, respectively [53].

## Results

### Study Population

On average, female patients affected by hypothyroidism in our study were 42.6 (SD 10.7) years old. The youngest participant was 20 years old, while the oldest was 71 years old. In the context of participants’ hypothyroid disease, the vast majority of participants (n=262, 82.4%) reported fatigue as a symptom, whereas only 18.9% (n=60) reported irregular menstruation as a symptom. Table 1 presents a complete description of the study population. Participants reported moderate physical health (mean 5.45, SD 1.97; range 0‐10), mental health (mean 5.86, SD 2.45; range 0‐10), and quality of life (mean 5.94, SD 2.23; range 0‐10).

In terms of eHealth, participants reported high levels of eHealth literacy (mean 31.85, SD 6.22; range 8‐40) and high digital confidence (mean 4.13, SD 0.93; range 1‐5), whereas internet anxiety (mean 1.59, SD 0.70; range 1‐5) was low, and digital overload was moderate (mean 2.56, SD 0.96; range 1‐5).

**Table 1.** Sample characteristics of female patients affected by hypothyroidism in a cross-sectional study conducted between April 2023 and April 2024 in Germany*.*

Characteristics	Participants (N=318), n (%)
Marital status	
Single	67 (21.1)
In a relationship	78 (24.5)
Married	147 (46.2)
Divorced or separated	19 (6)
Widowed	4 (1.3)
Other	3 (0.9)
Educational level	
No or lower secondary education or other	23 (7.2)
Higher secondary education	79 (24.8)
Higher education entrance qualification	105 (33)
University education	111 (34.9)
Occupational status	
Student	21 (6.6)
Nonworking	8 (2.5)
Sick leave	14 (4.4)
Part-time employed	89 (28)
Full-time employed	157 (49.4)
Retired	13 (4.1)
Other	16 (5)
Place of residence (population size)	
Large city (>100,000 residents)	98 (30.8)
Medium-sized city (>20,000 residents)	89 (28)
Small town or rural area (<20,000 residents)	131 (41.2)
Symptoms	
Fatigue	262 (82.4)
Weight gain	219 (68.9)
Depressed mood	218 (68.6)
Poor concentration	195 (61.3)
Dry skin	185 (58.2)
Loss of libido	165 (51.9)
Increased sensitivity to cold	155 (48.7)
Hair loss	140 (44)
Constipation	82 (25.8)
Low pulse or low blood pressure	64 (20.1)
Irregular menstruation	60 (18.9)
Hormone replacement therapy	289 (90.9)
Side effects of the hormone replacement therapy	31 (9.1)
Surgical operations	26 (8.2)

### Acceptance of mHealth Apps

Overall, the acceptance of mHealth apps among female patients affected by hypothyroidism was high (mean 4.10, SD 0.91; range 1‐5). More precisely, 76.1% (n=242) of participants reported high acceptance, 18.6% (n=59) reported moderate acceptance, and only 5.3% (n=17) reported low acceptance.

Group comparisons showed differences in acceptance depending on the place of residence (*F*_2,315_=3.66; *P*=.03; *f*=0.15). Posthoc group comparisons showed that participants living in a medium-sized city (>20,000 residents) reported significantly higher rates of acceptance than those living in big cities (>100,000 residents; *t*_315_=−26; *P*_adj_=.04; *d*=0.23).

Participants who reported fatigue as a symptom of their hypothyroid disease reported higher levels of acceptance than ones who did not report fatigue (*t*_316_=−3.56; *P*_adj_<.001; *d*=0.53). However, differences in acceptance were neither dependent on marital, educational, or occupational status nor dependent on undergoing hormone replacement therapy or surgical operations.

### Predictors of Acceptance of mHealth Apps

Multiple hierarchical regression analysis was performed to determine predictors of the acceptance of mHealth apps to improve the quality of life of female patients affected by hypothyroidism. First, sociodemographic data were included (*R*^2^=.034; *R*^2^_adj_=.015; *F*_6,31_=1.83; *P*=.09). Place of residence: medium-sized city (>20*,*000 residents; β=0.34; *P*=.02) and small town or rural area (<20,000 residents; β=0.28; *P*=.003) were significant predictors of acceptance. The explained variance of the first step was 3.4%.

Medical data were included in the second step (*R*^2^=0.086; *R*^2^_adj_=0.056; *F*_10,307_=2.88; *P*=.002). That step increased the explained variance significantly to 8.6% (∆*R*^2^=0.052; *F*_4,307_=8.85; *P*<.001). Fatigue (β=0.54; *P*<.001) emerged as a significant predictor of acceptance. In the third step, eHealth data (*R*^2^=0.013; *R*^2^_adj_=0.09; *F*_14,303_=3‐25; *P*<.001) significantly increased the explained variance to 13% (∆R^2^=0.044; *F*_4,303_=7.63; *P*<.001). Internet anxiety (β=−0.20; *P*=.002) was a significant predictor of acceptance.

In the final step, the three UTAUT predictors—EE, PE, and SI—were included (*R*^2^=0.561; *R*^2^_adj_=0.537; *F*_17,300_=22.62; *P*<.001). Explained variance of the final increased significantly to 56.1% (∆*R*^2^=0.431; *F*_3,300_=98.42; *P*<.001). EE (β=0.37; *P*<.001), PE (β=0.32; *P*<.001), and SI (β=0.20; *P*<.001) were significant predictors. Table 2 contains the final UTAUT model of acceptance and its predictors.

**Table 2.** Hierarchical regression model of acceptance of mHealth^a^ apps among female patients affected by hypothyroidism in a cross-sectional study conducted between April 2023 and April 2024 in Germany (N=318)^b^.

Predictors	B	β	*t* test *(df)*	*R* ^2^ ^c^	∆*R*^2^^d^	*P* value
Intercept	.88	−0.18	−0.97			.33
Step 1: sociodemographic data				0.034	0.034	
Age	.00	0.03	0.61			.54
Educational level (reference: no or lower secondary education or other)						
Higher secondary education	.02	0.03	0.16			.87
Higher education entrance qualification	−.15	−0.17	−1.04			.30
University education	−.00	−0.01	−0.03			.97
Place of residence (population size; reference: large city; >100,000)						
Medium-sized city (>20,000)	.06	0.07	0.68			.49
Small town or rural area (<20,000)	.03	0.04	0.41			.68
Step 2: medical data				0.086	0.052	
Physical health	.02	0.04	0.78			.44
Mental health	−.00	−0.00	−0.02			.98
Quality of life	−.02	−0.05	−0.97			.33
Fatigue	−22	0.24	2.18			.03
Step 3: eHealth-related data				0.13	0.044	
eHealth literacy	−.00	−0.02	−0.38			.70
Digital confidence	−.00	0.00	0.07			.95
Digital overload	−.01	−0.01	−0.20			.84
Internet anxiety	−.10	−0.08	−1.75			.08
Step 4: UTAUT^e^ predictors				0.561	0.431	
EE^f^	.40	0.37	8.04			<.001
PE^g^	.32	0.32	6.69			<.001
SI^h^	.19	0.20	4.38			<.001

a mHealth: mobile health.

b In Steps 2, 3, and 4, only the newly included variables are presented.

c *R*²: determination coefficient.

d ∆*R*2: changes in *R*2.

e UTAUT: unified theory of acceptance and use of technology.

f EE: effort expectancy.

g PE: performance expectancy.

h SI: social influence.

## Discussion

### Principal Findings

The purpose of this study was to determine the acceptance of mHealth apps, along with the factors influencing it, among female patients affected by hypothyroidism, with the aim of improving quality of life. Due to the reduced quality of life from its physical and mental symptoms and challenges in therapy (eg, medication adjustment), the results of this study may contribute to improved care for female patients affected by hypothyroidism. The overall acceptance of mHealth was high; more than three-quarters (n=242, 76.1%) of participants reported high acceptance, while 18.6% (n=59) reported at least moderate acceptance. Compared to similar studies representing different populations of patients with other chronic diseases [45-48], acceptance among female patients affected by hypothyroidism was high, which may result in higher utilization in practical implementation.

Acceptance of mHealth was especially high among patients affected by fatigue, which was also identified as a predictor of acceptance. Fatigue was a common symptom of participants in this study and is generally common in overt hypothyroidism [54,55]. Added to its frequency, fatigue as a symptom of various diseases is primarily associated with a reduction in quality of life [55-57]. As a result, mHealth interventions may contribute to managing symptoms by enhancing understanding and ways of coping with symptoms of fatigue. Regarding physical and mental health, no significant predictor of acceptance was identified, contrary to other studies [45-48]. However, participants reported only moderate levels of physical and mental health. This suggests an unmet need for additional health care offerings, for example, via mHealth interventions.

In regard to sociodemographic characteristics, living in a small or medium-sized city emerged as a significant predictor of acceptance. This relationship might be explained by a potentially lower availability of health care facilities and self-help groups in less populated areas. In response, mHealth interventions could address the lack of health care services and provide health care for patients in less densely populated areas. This interpretation is supported by previous studies, which also reported higher mHealth acceptance in less urbanized settings [46,58]. Unlike other studies, acceptance was not significantly predicted by age [45,47,48,52,59]. This finding is plausible given the growing tendency for older adults to use digital media, which might likely reflect the trend in the future [60]. Overall, the level of acceptance was also high among older female patients affected by hypothyroidism, suggesting that mHealth interventions represent a viable option in this patient collective. Moreover, unlike in previous studies, educational status did not emerge as a significant predictor of acceptance [52,61,62]. It should be noted, however, that 67.9% (n=216) of patients surveyed had at least a higher education entrance qualification or even university education, whereas only 7.2% (n=23) had no or lower secondary education. That phenomenon is commonly observed in research on digital health care [61,62] and suggests that mHealth interventions should be adapted to the needs of less-educated patients.

Concerning eHealth-related data, internet anxiety emerged as an important predictor of acceptance. Overall, levels of internet anxiety were low, which can be partly explained by the fact that the survey was conducted and completed entirely online. However, higher levels of internet anxiety were a significant negative predictor of acceptance. Thus, internet anxiety seems to represent a barrier to using mHealth interventions, which should be taken into account when designing and implementing such interventions [38,40], including concerns about privacy, trust in online information, and smartphone access [29,30]. Internet anxiety also primarily affects older adults [63,64], although a positive trend may be expected in this regard given the increasing number of older smartphone users [60]. Finally, the level of digital confidence and eHealth literacy was high but was not a significant predictor of acceptance, which is contrary to other studies [45,47,48].

The UTAUT model, which aims to predict users’ intentions to use an mHealth intervention and their actual use behavior [36], contributed significantly to the explained variance, and the 3 UTAUT predictors (ie, EE, PE, and SI) emerged as significant predictors of acceptance. Those findings are in line with the results of other studies [48,50,65-68]. Of all predictors identified, EE was the most significant predictor of acceptance. Accordingly, acceptance depends on the expected effort involved in interacting with the mHealth intervention. To maximize EE, developers should prioritize intuitive navigation, clear and minimalistic user interfaces, and personalization options that accommodate users with varying levels of digital literacy [69]. Tutorial features and onboarding processes, as well as automated reminders, may further lower the perceived effort required to use the app. A further significant predictor of acceptance was PE, which highlights the importance of ensuring that patients are convinced of the benefits of the intervention. Integrating evidence-based educational content, tracking tools for symptoms and treatment adherence, and feedback mechanisms may help patients recognize the value of using the app [70]. Allowing users to personalize content and adapt app features to their individual needs may further enhance both PE and EE. Finally, SI was a significant predictor of acceptance. Incorporating peer-support features (such as forums or patient communities), enabling positive feedback from health care professionals within the app, and integrating testimonials or success stories can encourage sustained engagement of SI. Partnerships with clinicians who recommend or prescribe the use of validated mHealth tools may further strengthen SI.

For future research, it would first be important to develop and validate a user-centered mHealth app specifically customized for this patient group. Actively involving patients in the design process and testing the app in terms of usability and perceived usefulness would provide a solid foundation for broader implementation. Subsequently, longitudinal studies tracking actual app use would be valuable to examine how initial acceptance translates into sustained engagement and real-world behavioral outcomes. This would also help to further address the intention-behavior gap and support the development of more effective mHealth interventions for patients with hypothyroidism.

In conclusion, this study highlights key factors that should be considered when designing mHealth interventions for female patients affected by hypothyroidism. In particular, EE, PE, and SI emerged as central predictors of acceptance. Ensuring intuitive and accessible design, clearly communicating the benefits of using the app, and fostering support from health care professionals may therefore promote higher acceptance and sustained engagement. Moreover, barriers such as internet anxiety should be addressed to ensure equitable access. Theoretically, this study expands the application of the UTAUT model to this patient group. By demonstrating the model’s explanatory power in this gender-specific chronic disease context, our findings contribute to advancing gender-sensitive mHealth research and underscore the importance of considering sociodemographic and health and mHealth-related factors in technology acceptance studies.

### Limitations

When discussing and interpreting our results, for example, for practical implications, the following limitations should be taken into account. First, the questionnaire was only available via internet access, and the diagnosis of hypothyroidism could not be objectively verified, as it is based on self-report. Along those lines, it should also be noted that some participants were recruited online, for example, via online self-help groups and social media, which might have led to a selection bias. Younger patients, accordingly, and participants with a higher level of digital confidence or less internet anxiety may have been overrepresented. While older adults were not actively excluded, the digital format required a minimum level of digital literacy and internet access, which may have limited their participation. However, as digital confidence among older adults continues to increase [71], future research should specifically target this group and individuals facing digital barriers. Second, the intention-behavior gap needs to be considered. Accepting an mHealth intervention leads to a higher intention to use it but does not necessarily result in actual use and behavior to the same degree [72,73]—the actual use should therefore be investigated in future studies. In addition, the generalizability of our findings is limited in several aspects. On the one hand, male patients were not included in this study: not only are female patients significantly more likely to be affected by hypothyroidism [5,6], but similarly designed studies have also shown that these questionnaires are less likely to be completed by male patients [46-48]. However, gender-specific differences in technology acceptance and the experience of hypothyroidism may influence outcomes in this group, and it remains unclear whether similar acceptance samples would emerge in male patients [74]. On the other hand, the sample was predominantly well-educated, which may have contributed to higher levels of digital confidence and mHealth acceptance. However, patients with lower educational levels are less likely to participate in eHealth studies and may face additional barriers that need further investigation [75-77]. Finally, as the study was conducted in a Western, German-speaking context, the findings may not directly translate to non-Western health care settings, where cultural factors, health system structures, and technology access may affect mHealth acceptance differently [78-80]. Future research should explicitly address these aspects to broaden the evidence base.

### Conclusions

Altogether, this study showed a generally high level of acceptance regarding mHealth apps to improve the quality of life among female patients affected by hypothyroidism, particularly among those experiencing fatigue. Several predictors of acceptance were identified as well: Along with the UTAUT predictors EE, PE, and SI, not living in a highly populated city was a driver of acceptance. Internet anxiety was found to be a barrier to using mHealth apps. By addressing key acceptance factors, mHealth interventions have the potential to significantly enhance the quality of life of female patients with hypothyroidism.

## Supplementary material

10.2196/67317 Checklist 1 Strengthening the Reporting of Observational Studies in Epidemiology (STROBE) Standards checklist.

## Abbreviations

BI: behavioral intention
EE: effort expectancy
mHealth: mobile health
PE: performance expectancy
SI: social influence
STROBE: Strengthening the Reporting of Observational Studies in Epidemiology
UTAUT: unified theory of acceptance and use of technology
